# Misdiagnosis of rivaroxaban-associated multiple intracranial hemorrhages as intracranial metastatic tumor: a case report

**DOI:** 10.3389/fmed.2026.1729651

**Published:** 2026-02-09

**Authors:** Fabao Xu, Qisheng Liu, Shunde Hua, Shaochun Yang

**Affiliations:** 1 The First Clinical Medical College, Gannan Medical University, Ganzhou, Jiangxi, China; 2 Department of Neurosurgery, The First Affiliated Hospital of Gannan Medical University, Ganzhou, Jiangxi, China

**Keywords:** atrial fibrillation, intracranial hemorrhage, intracranial metastatic tumor, magnetic resonance imaging, rivaroxaban

## Abstract

Rivaroxaban, a direct oral anticoagulant (DOAC), has emerged as a first-line therapy for the prevention and treatment of thromboembolic diseases, such as stroke in atrial fibrillation, due to its consistent anticoagulant effect. Nevertheless, vigilance remains imperative concerning its potential adverse reactions, particularly rare imaging manifestations that may result in misdiagnosis. This report details an 88-year-old female patient with atrial fibrillation who experienced multiple intracranial hemorrhages approximately 20 days after commencing rivaroxaban therapy. Initial contrast-enhanced cranial magnetic resonance imaging revealed multiple intracranial nodules with ring enhancement and edema, leading to a misdiagnosis of “multiple intracranial metastatic tumors with hemorrhage.” Following the discontinuation of rivaroxaban, no antitumor therapy was administered; only antihypertensive therapy and symptomatic management were provided. Dynamic follow-up revealed gradual lesion shrinkage, with partial resolution several months post-discontinuation. The case was ultimately diagnosed as rivaroxaban-associated multiple intracranial cerebral hemorrhages. This case highlights that rivaroxaban-associated intracranial hemorrhage may present as rare multiple nodular lesions. Their imaging characteristics can easily be confused with those of intracranial metastatic tumors. Clinical diagnosis requires comprehensive evaluation incorporating medication history, dynamic imaging follow-up, and lesion progression to avoid misdiagnosis and overtreatment. Additionally, for elderly patients with multiple comorbidities, the prescription of DOACs should include individualized evaluation to optimize dosage selection and monitoring strategies.

## 1 Introduction

Atrial fibrillation (AF) is one of the most common arrhythmias worldwide, with its prevalence increasing significantly with age, making it a major public health issue among the elderly population (1). Due to turbulent blood flow within the atria, AF patients are prone to thrombus formation. Research by Singer et al. indicates that age is an independent risk factor for increased thromboembolic risk in AF patients. Data show that the risk of thromboembolic events increases by 2.38-fold, 4.46-fold, and 8.14-fold in patients aged 65–74 years, 75–84 years, and ≥85 years, respectively (2). Anticoagulant therapy effectively reduces the risk of thromboembolic events by preventing thrombus formation. Compared to traditional vitamin K antagonists, direct oral anticoagulants (DOACs) like rivaroxaban act directly by inhibiting factor Xa (FXa). This mechanism not only reduces drug interactions but also eliminates the need for routine coagulation monitoring and strict dietary restrictions required with warfarin, leading to their widespread clinical adoption (3). Intracranial hemorrhage is one of the most serious adverse reactions of rivaroxaban, with an incidence lower than that of traditional vitamin K antagonists. Data from the ROCKET AF randomized double-blind trial demonstrated that the annual incidence of intracranial hemorrhage was approximately 0.5% per year in the rivaroxaban treatment group, compared with 0.7% per year in the warfarin control group, the hazard ratio (HR) for intracranial hemorrhage with rivaroxaban relative to warfarin was 0.67 (*p* = 0.02), indicating a statistically significant difference (4). According to previous literature reports, the imaging findings of rivaroxaban-related intracranial hemorrhage exhibit certain regular patterns: they are predominantly isolated cerebral parenchymal hematomas, while multiple nodular lesions with ring enhancement are extremely rare. The predilection sites are mostly the lobes of the cerebral hemispheres (predominantly the parietal and temporal lobes) and the deep gray matter nuclei (including the basal ganglia, thalamus, and other regions). Mild perilesional edema is often observed adjacent to the hematoma, and the risk of hematoma expansion is lower compared with warfarin-related intracranial hemorrhage (5). However, the present case is characterized by multiple nodular lesions with ring enhancement, whose imaging features are highly analogous to those of intracranial metastases, particularly hemorrhagic metastases. This represents the primary cause of misdiagnosis, and such cases have rarely been reported in national and international literature. By elaborating on the patient’s diagnostic and therapeutic process, the predisposing factors for misdiagnosis, and the optimization strategies for the clinical application of direct oral anticoagulants in elderly patients, this study combines the latest clinical evidence and guidelines to provide a reference for clinical practice. Additionally, it supplements the clinical data on rare hemorrhagic manifestations associated with rivaroxaban.

## 2 Clinical data

**Figure 1:**
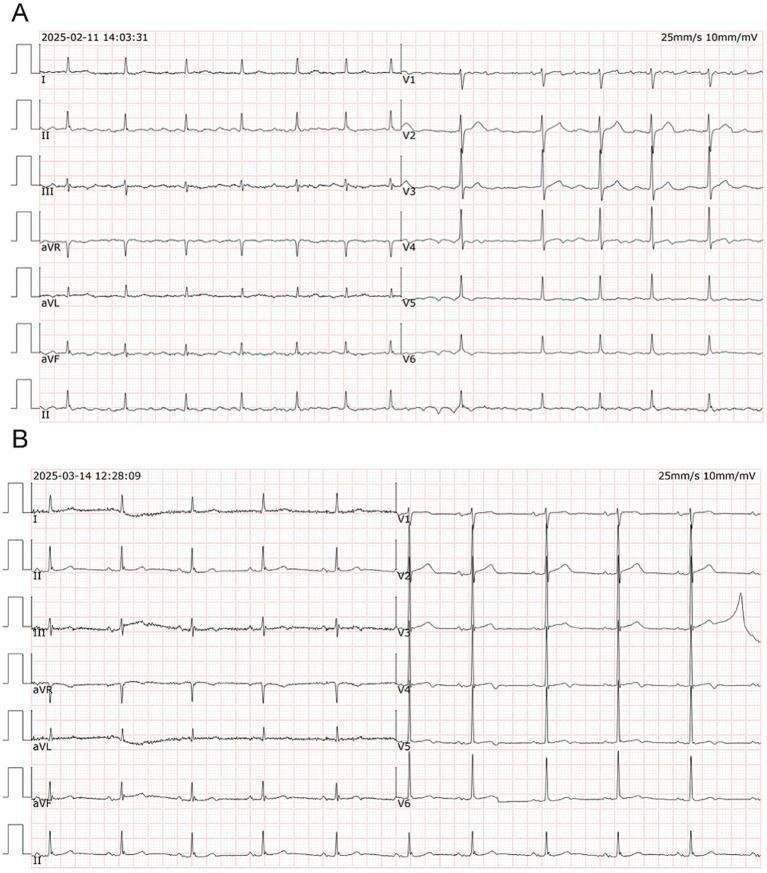
Electrocardiogram (ECG) on February 11 **(A)** indicates atrial fibrillation; ECG on March 14 **(B)** indicates sinus rhythm.

An 88-year-old female patient was admitted on March 14, 2025, presenting with “communication difficulties for over 10 days and the discovery of an intracranial mass 4 days prior.” The patient had no familial tumor or genetic disorder history and no smoking or alcohol consumption history. With excellent treatment compliance, she discontinued rivaroxaban per physician guidance, took antihypertensive drugs regularly, and completed all scheduled imaging follow-ups after discharge on March 21, 2025, with no new adverse events reported during the follow-up period. She had a history of hypertension for 10 years, controlled with long-term oral telmisartan 80 mg once daily; she had a history of coronary artery disease, but no anticoagulant medications were prescribed for coronary artery disease; she was hospitalized for cerebral infarction over 20 years ago and in May 2024, with no sequelae or history of cerebral hemorrhage. On February 11, 2025, she presented with “slurred speech, facial asymmetry with bilateral limb weakness for 30 min,” and was diagnosed with acute cerebral infarction and treated with alteplase thrombolysis. During hospitalization post-thrombolysis, ECG revealed atrial fibrillation (Figure 1A), prompting initiation of oral rivaroxaban 15 mg once daily for anticoagulation. At the time of readmission, she had been receiving the aforementioned rivaroxaban therapy for approximately 20 days.

**Figure 2:**
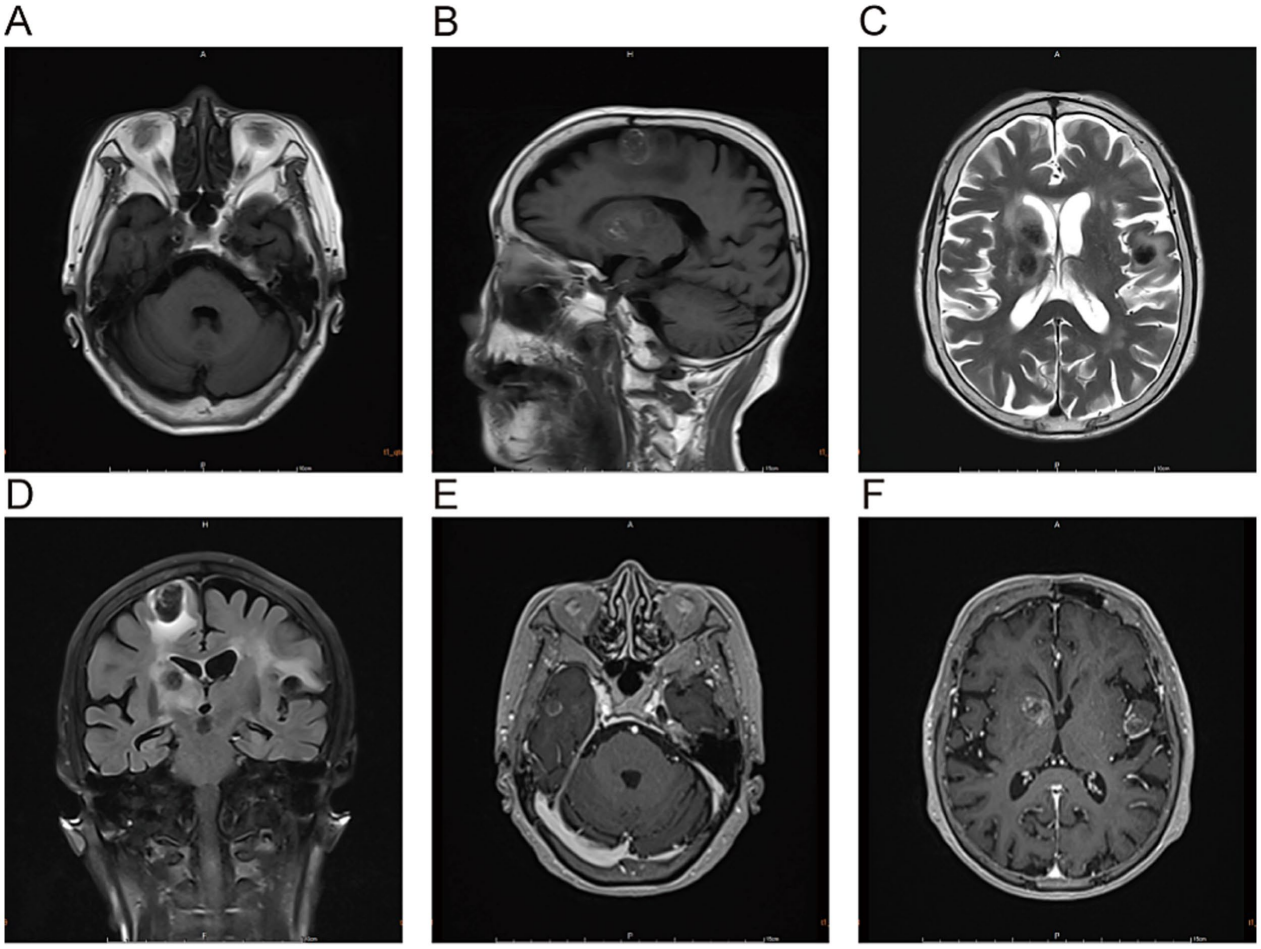
The lesions in the right frontal-temporal lobe and basal ganglia region, as well as the left frontal lobe, predominantly exhibit high signal intensity on T1-weighted images **(A,B)** and low signal intensity on T2-weighted images **(C)**. T2-FLAIRE sequences **(D)** reveal patchy perilesional edema surrounding the lesions. Contrast-enhanced scans **(E,F)** demonstrate ring enhancement.

**Figure 3:**
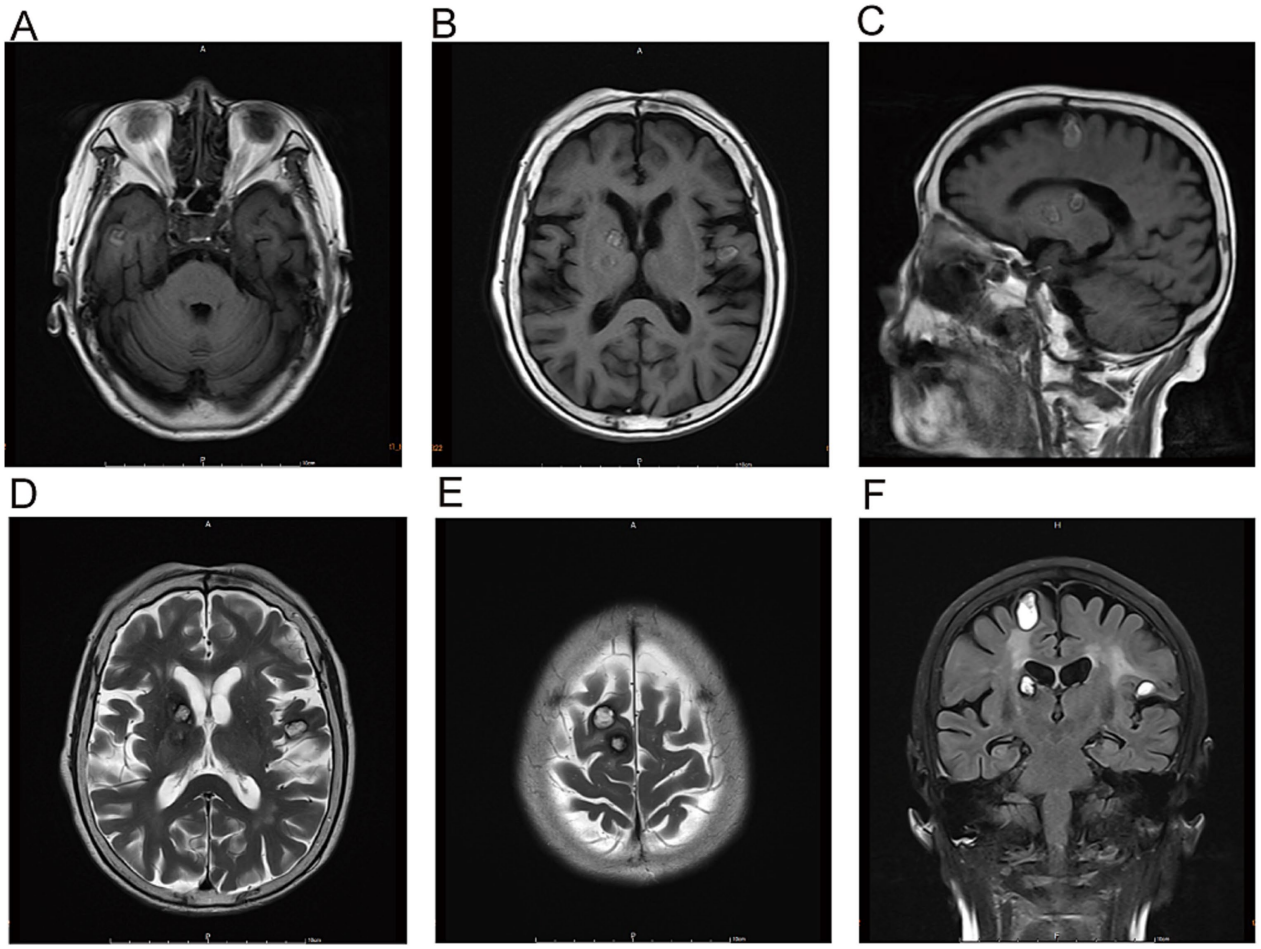
Multiple lesions are noted in the right frontal-temporal lobe, basal ganglia, and left frontal lobe, with perilesional edema. Some lesions have decreased in size compared to previous findings, and the edema has partially resolved. On March 30, the MRI scans demonstrated multiple lesions and perilesional edema in the right frontotemporal lobe, basal ganglia, and left frontal lobe. Specifically, the lesions and edema were partially reduced compared with previous findings on T1-weighted images (Panels **A–C**), T2-weighted images (Panels **C, D**), and T2-FLAIR sequence (Panel **F**).

**Figure 4:**
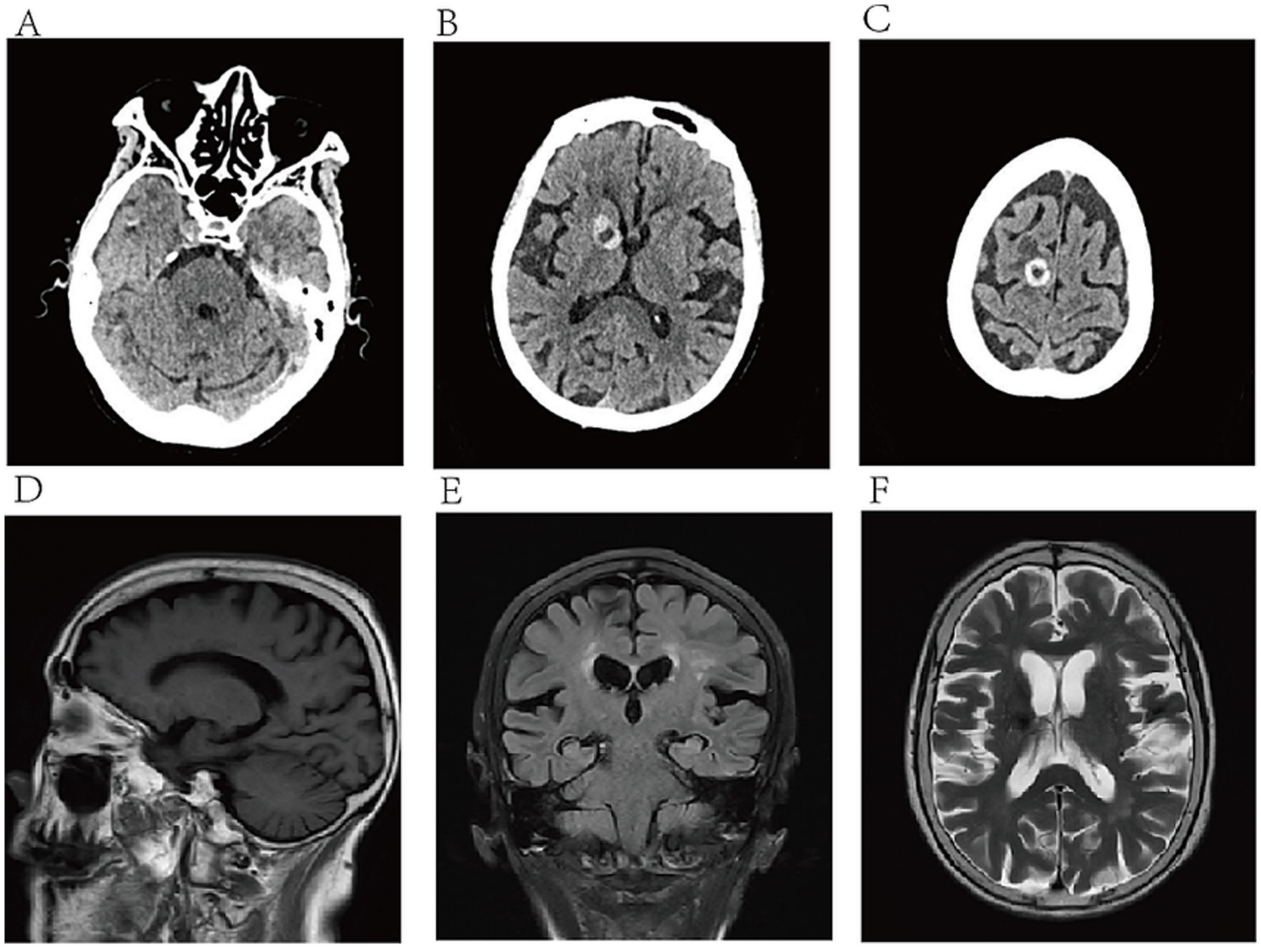
**(A–C)** CT scans obtained on March 30 revealed multiple intracranial nodular lesions; **(D–F)** MRI scans acquired on October 6 showed that most intracranial lesions had decreased in size compared with prior images, with some completely resolved.

Admission Physical Examination: BP: 152/69 mmHg. No palpable enlarged superficial lymph nodes throughout the body. She was alert and oriented. Bilateral limb muscle strength was grade V. Muscle tone was normal. Physiological reflexes were present. Pathological signs were negative. Laboratory Tests: Prothrombin time (PT) 12.3 s, Activated partial thromboplastin time (APTT) 34.8 s, International Normalized Ratio (INR) 1.04, Platelet count 205 × 10^9^/L—all of which were within normal limits. Serum creatinine 88 μmol/L, estimated glomerular filtration rate (eGFR) 59 mL/min/1.73 m^2^; serum albumin 3.62 g/dL (normal range: 4.00–5.50 g/dL); non-small cell lung cancer (NSCLC)-associated antigen CYFRA 21-1: 5.23 ng/mL (normal range: 0–3.3 ng/mL); pro-gastrin-releasing peptide (ProGRP): 118.00 pg./mL (normal range: 0–63 pg./mL); all other tumor markers were within normal limits. On March 10, 2025, cranial magnetic resonance imaging (MRI) with contrast (Figure 2) revealed multiple nodular mixed signal lesions in the right frontal-temporal lobe and basal ganglia, as well as the left frontal lobe, surrounded by patchy edema. Ring enhancement was observed on contrast-enhanced scans. Based on these findings, the preliminary diagnosis was “Possible brain metastases with hemorrhage.” Since our institution does not routinely perform diffusion-weighted imaging (DWI) and susceptibility-weighted imaging (SWI) sequences in clinical practice, this MRI examination was conducted using only T1-weighted, T2-weighted, fluid-attenuated inversion recovery (FLAIR), and contrast-enhanced sequences. Chest and abdominal computed tomography (CT) revealed bilateral multiple tiny pulmonary nodules and focal thickening of the gastric wall. After consultation with the Department of Respiratory Medicine, clinicians deemed these tiny pulmonary nodules benign proliferative nodules, with a recommendation for annual routine follow-up. No atypical cells were identified on gastroscopic pathological examination. On the patient’s second admission on March 14, 2025, electrocardiography (ECG) indicated a sinus rhythm (Figure 1B). A follow-up brain MRI scan performed on March 18, 2025, showed no significant changes in the size, signal intensity, or enhancement pattern of the lesions. Repeat brain MRI (Figure 3) and CT (Figures 4A–C) on March 30, 2025, demonstrated a reduction in the size of the lesions compared with prior images, with resolution of the surrounding edema. A further follow-up brain MRI on October 6, 2025 (Figures 4D–F) showed that most lesions had significantly decreased in size, and some had completely resolved.

Based on imaging and tumor marker results, the initial diagnosis was “intracranial metastatic tumors with hemorrhage.” Rivaroxaban was discontinued starting on March 11, 2025, to prepare for an upper gastrointestinal endoscopy. Following admission, the patient received daily dexamethasone to alleviate peritumoral edema, in addition to supportive treatments including blood pressure control, oxygen therapy, and fluid replacement. A follow-up cranial MRI on March 18, 2025 (7 days after rivaroxaban discontinuation) showed no significant changes in lesion size, signal intensity, or enhancement pattern, maintaining the diagnosis of metastatic tumors with hemorrhage. Due to the patient’s advanced age and the unidentified primary tumor site, the family declined surgery and biopsie. On March 21, 2025, the patient’s communication difficulties improved and mental status stabilized, leading to discharge. Subsequently, serial imaging follow-up examinations after discharge demonstrated the gradual resolution of the intracranial lesions, leading to the final definitive diagnosis of rivaroxaban-induced multiple intracranial hemorrhage. The overall timeline is summarized in Table 1.

**Table 1:** Timeline of the presented case.

Date	Examination findings	Treatment interventions
February 11, 2025	Acute cerebral infarction episode; electrocardiogram (ECG) demonstrated atrial fibrillation; coagulation function within normal limits; repeat head CT showed no evidence of intracranial hemorrhage	Alteplase thrombolysis; initiated oral anticoagulation with rivaroxaban 15 mg/day
March 4, 2025	Developed communication deficits; no focal limb motor impairment	No specific intervention
March 10, 2025	Contrast-enhanced brain MRI revealed multiple nodular lesions with ring enhancement, suspected of intracranial metastases with concurrent hemorrhage; abdominal CT showed focal thickening of the gastric body wall	Discontinued rivaroxaban; planned elective esophagogastroduodenoscopy (EGD)
March 14, 2025 (Admission)	Blood pressure: 152/69 mmHg; serum albumin: 3.62 g/dL; creatinine clearance (CrCl): 29.95 mL/min; CYFRA 21-1: 5.23 ng/mL; pro-gastrin-releasing peptide (ProGRP): 118.00 pg./mL	Telmisartan 80 mg/day for blood pressure control; dexamethasone 10 mg/day for perilesional edema reduction; switched to 5 mg/day after 4 days, and dexamethasone was discontinued on March 18
March 18, 2025	Follow-up brain MRI showed no significant changes in lesion size, signal intensity, or enhancement pattern	Continued symptomatic treatment
March 21, 2025 (Discharge)	Improved communication deficits; enhanced mental status	Anticoagulation not reinstated; continued blood pressure control
March 30, 2025	Combined brain MRI and CT revealed reduced lesion volume and complete resolution of surrounding edema	No specific treatment measures
October 6, 2025	Follow-up brain MRI showed marked reduction in most lesions, with complete resolution of some	No specific treatment measures

## 3 Discussion

This patient experienced multiple intracranial hemorrhages as a result of the combined effects of individual risk factors and the pharmacokinetics of rivaroxaban, specifically: 1. Rivaroxaban is a highly protein-bound drug, with 92–95% binding to plasma proteins, primarily albumin. A 2020 study by Wojakowski et al. demonstrated an association between albumin levels and bleeding risk in patients treated with rivaroxaban, indicating that patients who experienced bleeding events had significantly lower mean albumin levels during rivaroxaban therapy compared to non-bleeding patients, furthermore, each 1.0 g/dL decrease in albumin level was associated with a 4.4-fold increase in adjusted bleeding risk (6). Lower albumin levels at admission may increase free drug concentrations, which in turn enhance anticoagulant effects and increases bleeding risk. 2. Approximately one-third of rivaroxaban is metabolized renally. Clearance is significantly reduced in severe renal impairment, leading to elevated plasma drug concentrations. The Chinese Guidelines for the Management of Atrial Fibrillation recommend a dose of 20 mg once daily for non-valvular AF patients with creatinine clearance ≥50 mL/min and a dose of 15 mg once daily for those with 15–49 mL/min. However, Chinese researchers using a population pharmacokinetic-pharmacodynamic (PK-PD) model analysis of rivaroxaban found that exposure (AUC) in Chinese patients with a creatinine clearance ≥50 mL/min at a 15 mg dose is approximately equivalent to that in Caucasians at a 20 mg dose. For AF patients with a creatinine clearance <50 mL/min, dose reduction (e.g., 10 mg) is necessary to avoid excessive exposure (7). The patient’s creatinine clearance was approximately 29 mL/min (i.e., < 30 mL/min), placing her in the high bleeding risk population. Consistent with international guidelines, which recommend cautious use or switching to alternative anticoagulants in such cases, the administration of a daily dose of 15 mg of rivaroxaban resulted in increased drug exposure, which constituted the key predisposing factor for the hemorrhage. On admission, the patient’s platelet count and coagulation function indices, including prothrombin time, activated partial thromboplastin time, and international normalized ratio, were all within the normal range. Given the patient had no history of bleeding tendency and no use of antiplatelet agents, we ruled out intrinsic abnormalities of the coagulation system such as thrombocytopenia, platelet dysfunction, and congenital coagulation factor deficiency. However, due to the unavailability of anti-Xa factor activity assays and rivaroxaban plasma concentration measurements in our institution, we lacked objective evidence of drug accumulation—a major limitation of the present study.

First, regarding the selection of rivaroxaban, the core consideration was adherence in the elderly patient: an 88-year-old patient with limited cognitive function and self-management ability would benefit significantly from a once-daily oral regimen, which could markedly reduce the risk of missed or incorrect doses. This was the primary rationale for choosing rivaroxaban at that time. However, the critical high-risk factor of renal insufficiency was not fully integrated into the decision-making process, resulting in inherent limitations in the choice of anticoagulant. Notable pharmacokinetic differences exist between rivaroxaban and apixaban. Rivaroxaban is administered once daily, leading to substantial peak-trough fluctuations in plasma concentration and a relatively higher bleeding risk. In contrast, apixaban is given twice daily, which yields a more stable plasma concentration profile with lower peak-trough variability and minimal fluctuations. A study investigating plasma concentrations of rivaroxaban and apixaban in Asian patients with atrial fibrillation found that in the rivaroxaban group, only 33.8% of peak concentrations and 64.4% of trough concentrations fell within the expected target range—figures significantly lower than those in the apixaban group. Furthermore, subtherapeutic rivaroxaban concentrations were more common, particularly for peak levels. This indicates that due to its higher peak-trough variability, rivaroxaban may result in either excessively high peak concentrations or inadequately low trough concentrations in some patients, thereby simultaneously increasing the risk of both bleeding and thrombosis (8). In contrast, apixaban, with its more stable concentration profile, is likely to provide more predictable anticoagulant efficacy, which facilitates a better balance between bleeding and thrombotic risks. In the present case, the selection of apixaban instead of rivaroxaban could have reduced the bleeding risk through a more stable plasma concentration profile, making apixaban a more suitable choice that aligns with the patient’s individualized characteristics of advanced age and renal insufficiency. Second, diagnostic bias stemmed from the nonspecific interpretation of tumor markers. As documented in the literature, CYFRA 21-1—a biomarker associated with NSCLC—is not lung cancer-specific. Mild elevation of this marker can be linked to smoking, advanced age, and physiological fluctuations (9, 10). The mild CYFRA 21-1 elevation observed in the present patient was a nonspecific finding. Combined with the results of subsequent gastroscopy and colonoscopy, serial follow-up chest CT scans, and the resolution of intracranial lesions, the possibility of an underlying tumor can be definitively ruled out.

The initial diagnostic error was attributable to overreliance on a single tumor marker. Additionally, inherent limitations in the selection of imaging modalities further contributed to the misdiagnosis. When the patient first presented for evaluation on March 10, 2025, contrast-enhanced brain MRI was performed directly, without prior screening via brain CT. CT serves as the first-line imaging modality for acute intracerebral hemorrhage, enabling rapid identification of acute hyperdense hematomas. However, its sensitivity in depicting ring enhancement and the degree of perilesional edema are far lower than that of MRI. Conversely, MRI, especially susceptibility-weighted imaging (SWI) may overestimate the size and number of hemorrhages due to susceptibility artifacts. Nevertheless, even if an initial brain CT had been obtained in this case, it would only have detected multiple nodules, which would still have led to a misdiagnosis of hemorrhagic intracranial metastases. That said, a diagnostic strategy combining initial CT, full-sequence MRI, and dynamic serial follow-up can significantly improve the accuracy of differential diagnosis and circumvent the limitations inherent to a single imaging modality.

In terms of differential diagnosis, cerebral amyloid angiopathy (CAA) was excluded first, as its classic imaging manifestations differ substantially from those observed in the present case. The typical imaging feature of CAA is multiple cortical and subcortical microhemorrhages in the cerebral lobes, which present as small, round hypointense foci on SWI or T2-weighted sequences without ring enhancement (11). In contrast, the lesions in this patient were nodular with ring enhancement and gradually regressed after rivaroxaban discontinuation—features that are inconsistent with CAA, thus ruling out this diagnosis. Next, cerebral vasculitis was also excluded based on clinical and imaging discrepancies. Cerebral vasculitis is typically accompanied by clinical manifestations such as cognitive impairment, seizures, and focal neurological deficits. Its imaging findings include small cortical or subcortical infarcts or microhemorrhages, with hemorrhages mostly appearing as punctate or patchy lesions distributed in a pattern corresponding to vascular territories. This condition generally responds well to corticosteroid therapy (12, 13). However, the patient in this case had a poor response to dexamethasone administered during hospitalization; the lesions improved only after drug discontinuation and blood pressure control, with no relevant accompanying symptoms. Therefore, cerebral vasculitis was also excluded.

In studies designed to compare the efficacy and safety of early versus late initiation of DOACs in patients with atrial fibrillation-related acute ischemic stroke, some research has demonstrated that early DOAC initiation does not increase the risk of hemorrhagic transformation and may help reduce the recurrence of thromboembolic events. However, a critical caveat must be emphasized: the core context of these trials focused on “hemorrhagic transformation after ischemic stroke,” excluding primary intracranial hemorrhage and severe PH1 and PH2 types of hemorrhagic transformation. The underlying hemorrhagic mechanism in these trials was primarily attributed to increased vascular permeability caused by reperfusion of the ischemic region. This represents a fundamental pathological distinction from the primary intracranial hemorrhage in the present case, which was induced by rivaroxaban-mediated inhibition of coagulation factor Xa. Additionally, there was no evidence of acute ischemic stroke as the etiology of the current presentation in this patient, which means the case is not consistent with the study population enrolled in the ELAN trial. Therefore, the conclusions of this trial hold only indirect reference value for the diagnosis and treatment of the present case (14).

A recent meta-analysis demonstrated that the risk of intracranial hemorrhage associated with DOACs is significantly lower than that with low-molecular-weight heparin (LMWH) in patients with brain tumors (15). This advantage is particularly pronounced in those with primary brain tumors, whereas no statistically significant difference was observed in patients with metastatic brain tumors. In the present case, the patient was initially misdiagnosed with intracranial metastases. Even if the patient had indeed presented with a tumor, DOACs would still have been a safer anticoagulant option. This finding further supports the clinical utility of DOACs in complex patient populations. It should be noted that bridging therapy with LMWH was not administered to this patient. Instead, following thrombolysis for acute cerebral infarction on February 11, 2025, a repeat computed tomography (CT) scan on February 12, 2025, revealed no hemorrhagic transformation. Oral anticoagulation with rivaroxaban was therefore directly initiated at a dose of 15 mg/day. Regarding the resumption of anticoagulant therapy, the decision to restart anticoagulation after the acute phase of intracranial hemorrhage requires an individualized assessment, with a careful balance between the patient’s ischemic and hemorrhagic risks. Current evidence supports restarting anticoagulation in most patients within 2 to 16 weeks after the bleeding event. However, patients with lobar hemorrhage face a higher risk of rebleeding, and thus anticoagulation should be delayed until at least 4 weeks post-hemorrhage or even longer (16). In the present case, the patient was initially suspected to have “intracranial metastases complicated by hemorrhage” and had a HAS-BLED score of 3, prompting the recommendation to defer anticoagulation. Additionally, due to the patient’s advanced age and the family’s refusal of left atrial appendage occlusion (LAAO) therapy, anticoagulation was not resumed. Instead, strict blood pressure control was continued as the primary management strategy.

For elderly patients with multiple comorbidities (e.g., complicated by renal insufficiency, hypoalbuminemia, or a history of prior stroke), the following principles should be adhered to for the administration of DOACs: (1) For Chinese elderly patients with a creatinine clearance of < 30 mL/min, rivaroxaban is recommended to be dose-reduced to 10 mg once daily; alternatively, apixaban at 2.5 mg twice daily is preferred as the first-line agent. (2) Prior to medication initiation, laboratory tests including serum albumin level, renal function, coagulation function, and platelet function should be performed to systematically screen for high-risk factors for hemorrhage. (3) For institutions with available testing capabilities, regular monitoring of anti-Xa factor activity is recommended, with dynamic dose adjustment to avoid drug accumulation and thereby reduce the risk of bleeding.

The strengths of this study are as follows: it reports a rare imaging manifestation of rivaroxaban-related intracranial hemorrhage, supplementing relevant clinical data; it provides a detailed analysis of the causes of misdiagnosis and key considerations for the clinical application of DOACs in elderly patients, offering practical references for clinical practice; and it confirms the lesion outcome through long-term dynamic follow-up, ensuring high diagnostic reliability. However, the study also has several inherent limitations: first, it is a single case report, which restricts the generalizability of the research findings; second, the lack of histological biopsy, as well as the failure to measure anti-Xa factor activity and rivaroxaban plasma concentration, results in the absence of direct evidence of drug accumulation; third, the initial imaging evaluation did not include preliminary CT screening, leading to limitations associated with the choice of imaging modality.

## 4 Conclusion

Rivaroxaban-associated intracranial hemorrhage may present as rare multiple nodular ring-enhancing lesions, whose imaging features can be easily confused with those of intracranial metastatic tumors. The risk of misdiagnosis significantly increases, particularly when patients exhibit elevated tumor markers. Therefore, when patients on DOACs present with multiple intracranial lesions, a comprehensive assessment incorporating medication history, tumor screening results, and dynamic imaging evolution of the lesions is essential to avoid overtreatment due to misdiagnosis. Additionally, clinicians should exercise particular caution when prescribing medications to elderly patients with concomitant renal insufficiency or hypoalbuminemia. Rivaroxaban dosage should be strictly adjusted based on individual patients’ circumstances. Hospitals with the capacity should perform coagulation factor monitoring to dynamically assess coagulation function, thereby further reducing bleeding risks in such patients. Future studies with larger sample sizes are needed to validate the rare imaging phenotype of rivaroxaban-associated ICH and refine dosage strategies for elderly patients with comorbidities.

## Data Availability

The original contributions presented in the study are included in the article/supplementary material, further inquiries can be directed to the corresponding author.
